# Supplementary material for: Resolving the full spectrum of human genome variation using Linked-Reads
Source: Genome Res. 2019 Apr;29(4):635–45. doi: 10.1101/gr.234443.118 (PMC6442396; doi:10.1101/gr.234443.118)
Supplement: Supplemental Material [file supp_gr.234443.118_Supplemental_longranger-2.2.zip › longranger-2.2.tar.bz2!/lr2.2-public/lib/stan/cmdstan/stan/doc/api/html/dir_29b20cd43524c7f0fc77aef3c7e38f50.html]

Stan: src/stan/gm Directory Reference


|  |  |
| --- | --- |
|  | Stan  2.5.0  probability, sampling & optimization |


- Main Page
- Related Pages
- Namespaces
- Classes
- Files

All Classes Namespaces Files Functions Variables Typedefs Enumerations Enumerator Friends Macros Pages

- src
- stan
- gm

gm Directory Reference

|  |  |
| --- | --- |
| Directories | |
| directory | arguments |
|  | |
| directory | grammars |
|  | |

|  |  |
| --- | --- |
| Files | |
| file | ast.hpp [code] |
|  | |
| file | ast\_def.cpp [code] |
|  | |
| file | compiler.hpp [code] |
|  | |
| file | error\_codes.hpp [code] |
|  | |
| file | generator.hpp [code] |
|  | |
| file | parser.hpp [code] |
|  | |


---

[ Stan Home Page ]

*© 2011–2014,
Stan Development Team.*
